# Abnormal sleep blood pressure patterns are associated with the diffusion tensor imaging along the perivascular space index in cognitively impaired individuals

**DOI:** 10.3389/fnagi.2025.1578270

**Published:** 2025-08-29

**Authors:** Mariateresa Buongiorno, Gonzalo Sánchez-Benavides, Giovanni Caruana, Andrea Elias-Mas, Cristina Artero, Natalia Cullell, Pilar Delgado, Darly Milena Giraldo, Clara Marzal-Espí, Oriol Grau-Rivera, Alejandro de la Sierra, Ariane Delgado-Sanchez, Nicola J. Ray, Jerzy Krupinski

**Affiliations:** ^1^Neurology Department, Vall d'Hebron University Hospital, Barcelona, Spain; ^2^Neurovascular Diseases Research Group, Vall d'Hebron Research Institute, Barcelona, Spain; ^3^Barcelonaβeta Brain Research Center (BBRC), Pasqual Maragall Foundation, Barcelona, Spain; ^4^Hospital del Mar Research Institute, Barcelona, Spain; ^5^Centro de Investigación Biomédica en Red de Fragilidad y Envejecimiento Saludable (CIBERFES), Madrid, Spain; ^6^Radiology Department, Hospital Universitari Mútua de Terrassa, Terrassa/Barcelona, Spain; ^7^Institute for Research and Innovation Parc Taulí (I3PT), Sabadell, Spain; ^8^Genetics Doctorate Program, Universitat de Barcelona (UB), Barcelona, Spain; ^9^Department of Neurology, Fundació Assistencial Mútua Terrassa, Terrassa, Spain; ^10^Fundació per a Docència i Recerca, Mútua Terrassa, Terrassa, Spain; ^11^Internal Medicine Department, University Hospital Mutua de Terrassa, Terrassa, Spain; ^12^University of Barcelona, Terrassa, Spain; ^13^Department of Psychology, Faculty of Health and Education, Brooks Building, Manchester Metropolitan University, Manchester, United Kingdom; ^14^Department of Life Sciences John Dalton Building, Faculty of Science and Engineering, Manchester Metropolitan University, Manchester, United Kingdom

**Keywords:** Alzheimer's disease, blood pressure, DTI-ALPS, glymphatic, non-dipping

## Abstract

**Introduction:**

Blood pressure (BP) physiologically dips during sleep, and a lack of dipping is associated with adverse health outcomes and cognitive decline. Vascular pulsatility is the primary driver of glymphatic cerebrospinal fluid (CSF) transport, which removes metabolic waste products from the brain during sleep. We hypothesized that abnormal sleep BP patterns may affect glymphatic system health and that this relationship may result in lower diffusion tensor imaging along the perivascular space (DTI-ALPS) indices, a proposed neuroimaging index of glymphatic health.

**Methods:**

A total of 21 participants with mild-to-moderate cognitive impairment underwent 24-h ambulatory BP monitoring (ABPM), DTI-MRI, and Alzheimer's disease (AD) biomarker assessments. Of them, eight participants were classified as dippers (≥10%) and 13 as non-dippers (< 10%), using the sleep/awake systolic BP (SBP) percentage of change.

**Results:**

We found that the non-dippers had lower DTI-ALPS indices, even after adjusting for age and clinical stage (*p* = 0.013). Stiffness measures (pulse wave velocity) were negatively correlated with DTI-ALPS (*r* = −0.5), but the association disappeared after adjusting for age. Positive AD biomarkers were more frequently observed in the individuals who were classified as non-dippers for both systolic and diastolic BP (DBP), compared to the systolic and diastolic dippers (*p* = 0.041).

**Discussion:**

Our findings suggest that deviations from the physiological BP dipping sleep pattern may be related to poorer glymphatic function and increased AD pathology.

## Introduction

The circadian pattern of blood pressure (BP), as measured by 24-h ambulatory BP monitoring (ABPM), typically shows higher values during the day and a 10–20% reduction during sleep, a phenomenon known as “dipping” (Pickering, 1982; de la Sierra, 2024). The absence of this normal dipping pattern, referred to as non-dipping, has been associated with future adverse health outcomes, including increased cardiovascular risk (Salles et al., 2016; Lempiäinen et al., 2024) and cognitive decline (Gavriilaki et al., 2023). A recent systematic meta-analysis found that individuals with a normal dipping pattern had a 63% lower risk of all-cause dementia compared to non-dippers. Moreover, those classified as “risers” (non-dippers with even higher nighttime than daytime BP) were found to have a 6-fold increased risk of abnormal cognitive function compared to dippers (Gavriilaki et al., 2023). Notably, a longitudinal study over 24 years involving more than 1,500 Swedish older men found that the riser pattern was linked to an increased risk of being diagnosed with any form of dementia and Alzheimer's disease (AD) but not with vascular dementia (Tan et al., 2021). Such findings suggest the existence of mechanisms that may act as risk factors for AD, independent of direct cerebrovascular damage.

Studies focusing on patients already diagnosed with AD have revealed that abnormal ABPM patterns (non-dipper, riser, or extreme dipper) are highly prevalent (>80%) (Chen et al., 2013; Wang et al., 2021), compared to matched controls (38%) (Chen et al., 2013). Similarly, individuals with mild cognitive impairment (MCI), the transitional stage between intact cognition and dementia, seem to exhibit higher nighttime systolic BP (SBP) compared to controls (Riba-Llena et al., 2016), and the presence of MCI is more frequently observed in extreme dippers (32.0%), non-dippers (30.0%), and risers (50.0%) compared to dippers (13.2%) (Guo et al., 2010).

Research exploring the association between ABPM patterns and pathophysiological markers of AD is very limited. Tarumi et al. reported that among patients with amnestic MCI, those with a non-dipping pattern showed increased levels of amyloid-β (Aβ) deposition in the posterior cingulate, a key region in the early accumulation of AD pathology, as measured by the 18F-florbetapir PET SUVR (Tarumi et al., 2015).

One hypothesis for the association between abnormal sleep BP and cognitive decline revolves around the potential impact on the glymphatic system, a brain-wide waste clearance pathway that is primarily active during sleep. The glymphatic system relies heavily on vascular pulsatility, generated by the rhythmic contractions of the arteries, to drive cerebrospinal fluid (CSF) through perivascular spaces, facilitating the removal of metabolic waste products from the brain (Iliff et al., 2013; Nedergaard and Goldman, 2020). In 2018, Mestre et al. demonstrated that CSF flow is pulsatile and synchronized with the cardiac cycle, using particle tracking in live mice, with arterial wall motion, driven by perivascular pumping, as the primary mechanism. They used *in vivo* two-photon particle-tracking to show that every heartbeat deforms the walls of pial arteries, acting as a “perivascular pump” that drives CSF forward at 20 μm s^−1^, tightly phase-locked to the cardiac R-wave. The peak lateral motion of the wall (21 μm s^−1^) follows the heartbeat by just 30–40 ms, confirming that arterial pulsations are the primary driver of peri-arterial flow. When the authors acutely raised mean arterial pressure by 75% with intravenous angiotensin-II, the lumen diameter stayed constant; however, the stiffer wall produced a more asymmetric waveform. Short and sharper recoil phases frequently sent fluid backward. Quantitatively, back-flow events increased by 20%, and the net downstream speed fell by 40%, despite unchanged vessel caliber (Mestre et al., 2018). These results offer a clear mechanistic explanation for how hypertension, or any condition that alters arterial compliance, can impair glymphatic clearance. As cerebral arterial pulsatility is a major driver of CSF influx through the brain parenchyma and sleep plays a critical role, we hypothesize that deviations from the normal blood pressure pattern during sleep may plausibly impair the efficiency of waste clearance via the glymphatic pathway.

To the best of our knowledge, no direct evidence linking sleep BP patterns with glymphatic function has been established. This study aimed to investigate the relationship between dipping and non-dipping sleep BP patterns and diffusion tensor imaging along the perivascular space (DTI-ALPS), a postulated neuroimaging proxy for glymphatic function, in individuals with cognitive impairment. Our main hypothesis was that any deviation from the sleep dipping BP pattern would be associated with a lower DTI-ALPS index, suggesting poorer glymphatic functioning.

## Methods

### Participants

The sample was composed of 21 participants with cognitive impairment, recruited prospectively between December 2022 and December 2023 from the Cognition and Behavior Unit at the Department of Neurology, Hospital Universitari MútuaTerrassa, Barcelona, Spain. The study was offered to all patients meeting the inclusion criteria who attended the Unit for diagnostic evaluation. The inclusion criteria were as follows: male or female, aged between 60 and 80 years; minimum reading and writing capacity to be able to perform the cognitive impairment tests; a score of at least 0.5 in the memory domain of the Clinical Dementia Rating (CDR); and a diagnosis of either amnestic MCI or dementia. Additional details are provided in (Ray et al. 2024).

### Clinical measures

The participants were staged using the global CDR score. Global cognitive performance was assessed using the mini–mental state examination (MMSE), and episodic memory was evaluated using the age-adjusted delayed memory index of the Repeatable Battery for the Assessment of Neuropsychological Status (RBANS) (reference population mean = 100, SD = 15).

### Ambulatory BP data collection and definition of BP night patterns

ABPM was performed for 24 h using the BP monitor Mobil-O-Graph^®^ PWA (I.E.M. Industrielle Entwicklung Medizintechnik GmbH, Stolberg, Germany). The data were processed using the provided Hypertension Management Software Client-Server version 5.2.2 (HMS CS 5.2.2). The procedure was as follows: between 9:00 a.m. and 11:00 a.m., the participants arrived at the clinic, and the device was placed on the non-dominant arm. They were instructed to avoid efforts during BP measurements and to record any relevant incidents in the provided diary. They returned the morning after at approximately the same time. The device was removed, and the data were downloaded. The participants were instructed to record their sleep and wake times in the diary, as well as any incidents occurring during measurements, with assistance from their relatives if necessary. BP measurements were taken every 20 min over a 24-h period. Only valid readings were used in the computation of mean BP during awake and sleep periods. We averaged BP measurements based on the actual sleep and awake times reported in the diary, and we calculated the relative magnitude of BP changes from the awake to sleep period using the following formula:


$$\begin{array}{l} \text{Percentage of sleep BP dip}=\left(1-\frac{\text{mean BP sleep}}{\text{mean BP awake}}\right)\times \;100. \end{array}$$

Dipping patterns were defined according to current hypertension guidelines (O'Brien et al., 2013; Mancia et al., 2023). We categorized systolic BP (SBP) sleep patterns in two main categories: dipping (sleep SBP dip ≥10%) and non-dipping (sleep SBP dip < 10%). Extreme patterns were also labeled within each group: extreme dipping (sleep SBP dip >20%) and rising (sleep SBP dip < 0%).

Since we hypothesized that any deviation from the physiological patterns of sleep BP may be deleterious for glymphatic function, we also defined groups based on diastolic BP (DBP). For secondary analyses, we defined a normal sleep BP pattern as when both SBP and DBP exhibited a “dipping” pattern, and considered any deviation from “dipping” as an abnormal sleep BP pattern. Two groups within the abnormal patterns were created: “One Abnormal,” defined when only one abnormal pattern (either SBP or DBP different from dipping) was found, and “Two Abnormal,” when both SBP and DBP sleep patterns deviated from dipping.

Peripheral stiffness was explored using the mean of the augmentation index (AIx) corrected for a heart rate of 75 bpm (AIx@75). Central stiffness was measured using pulse wave velocity (PWV).

### MRI acquisition and DTI-ALPS calculation

Scans were acquired using a 3T MR scanner (Philips Ingenia Elition). A standardized MR protocol was used for the acquisition, including a diffusion-weighted imaging (DWI) sequence (TR = 2.53, TE = 0.07, slice thickness = 2.2 mm, voxel size = 1.69 × 1.69 x 2.2. DWI was performed in 128 directions (diffusion b = 1,000 s/mm^2^) and in one acquisition without diffusion weighting (B0). The DWI images were processed using FSL (Jenkinson et al., 2012). The images were skull stripped (Smith et al., 2004), and eddy current-induced distortions and subject movements were corrected (Andersson and Sotiropoulos, 2016). To calculate the ALPS index, FSL's DTIFIT was used to create diffusivity maps in subject space along the x, y, and z directions, as well as a color-coded vector image showing the principal diffusion tensor direction (V1). To identify the location of the perivascular space (PVS), SWI was spatially co-registered with the subject-space B0 image using ANTs and superimposed on the V1 image to identify the medullary veins located perpendicularly to the PVSs and within the projection and association fibers. A total of two 4 mm spheres were placed at these locations, and diffusion values in the x, y, and z directions were extracted and used to compute the ALPS index (Taoka and Naganawa, 2020) according to the following equation:


$$\begin{array}{l} \text{ALPS index}=\frac{\text{mean }\left(\text{Dxxproj},\text{ Dxxassoc}\right)}{\text{mean }\left(\text{Dyyproj},\text{ Dzzassoc}\right)}\; \end{array}$$

### AD biomarker status

The presence of AD pathology was defined as having a positive visual read on amyloid PET (^18^F-Flutemetamol), as rated by an experienced nuclear medicine physician following standard guidelines. For one participant without available PET data, we used the CSF ratio, t-tau/Aβ42, quantified using Lumipulse assay kits from Fujirebio (Fujirebio Inc. Europe, Gent, Belgium) and local cut-off values (Álvarez et al., 2018).

### Statistical analyses

We compared demographic, clinical, and AMBP variables and DTI-ALPS index scores between the dipping and non-dipping groups using the Mann–Whitney *U*-test for continuous variables and Fisher's exact test for categorical variables. To test our main hypothesis and explore differences in DTI-ALPS between the dipping and non-dipping groups, we performed a rank-based regression analysis, accounting for the effects of age and the CDR, using the Rfit package. Further adjustments for education were also made. As exploratory analyses, we also assessed the association between DTI-ALPS and continuous sleep SBP changes using correlational analyses.

In secondary exploratory analyses, we created three groups to better account for any deviations from the physiological sleep BP dipping pattern in both SBP and DBP. Any deviation from the normal dipping pattern (i.e., non-dipping, riser, or extreme dipping) was classified as abnormal. We also explored the differences among the Normal (dipping pattern in both SBP and DBP), One Abnormal BP (abnormal pattern either in SBP or DBP), and Two Abnormal BP (abnormal pattern in both SBP and DBP) groups using the Kruskal–Wallis test, with further adjustments for age. We also assessed the association between sleep, awake, and total Aix@75 and PWV measures—as proxies of vascular stiffness—and DTI-ALPS using correlational analyses.

A *p*-value threshold of < 0.05 was used to determine statistical significance for the main outcome. *P*-values < 0.1 were considered trends. Confidence intervals and effect sizes were also provided. Given the exploratory nature of the secondary outcomes, no adjustments for multiple comparisons were made. All analyses and plots were performed using the R statistical software.

## Results

### Prevalence of SBP dipping/non-dipping patterns and group differences

A total of 13 participants (62%) of the 21 recruited displayed a non-dipping sleep SBP pattern. Descriptive data for demographic, clinical, AMBP, and DTI-ALPS measures by dipping/non-dipping patterns are shown in Table 1. No differences in age, sex, CDR, and cognitive outcomes were found between the SBP dipping and non-dipping groups, although the non-dippers tended to have lower cognitive scores [MMSE, *p* = 0.1, ε^2^ = 0.09 CI 95% [−0.05, 0.50]] and a higher proportion of positive AD biomarkers (85% vs. 50%; *p* = 0.14). The dippers had a higher level of education [*p* = 0.04, ε^2^ = 0.17 CI 95% [0.05, 0.61]], lower mean sleep SBP measures [*p* = 0.02, ε^2^ = 0.69 CI 95% [0.44, 0.75]], and lower sleep PWV values [*p* = 0.03, ε^2^ = 0.19 CI 95% [0.04, 0.58]].

**Table 1:** Demographic, clinical, cognitive, BP, and DTI-ALPS descriptive statistics by sleep SBP patterns.

	**SBP dipping**	**SBP non-dipping**	***P***
Number of individuals	8	13	
Age, years (median [IQR])	72.5 [10.5]	74.0 [4.0]	0.44
Sex, female *n* (%)	5 (62.5%)	10 (76.9%)	0.63
Education, years (median [IQR])	11.5 [5.0]	7.0 [5.0]	**0.04**
MMSE (median [IQR])	28.0 [6.5]	21.0 [7.0]	0.10
RBANS delayed memory score (median [IQR])	75 [28.5]	54 [27]	0.24
**CDR global score**			0.71
CDR 0.5 *n* (%)	6 (75%)	11 (84.6%)	
CDR 1 *n* (%)	1 (12.5%)	2 (15.4%)	
CDR 2 *n* (%)	1 (12.5%)	0 (0%)	
AD biomarker status, positive *n* (%)	4 (50%)	11 (84.6%)	0.14
Hypertension, presence *n* (%)	3 (37.5%)	9 (69.2%)	0.20
BMI (median [IQR])	24.3 [9.2]	25.3 [3.8]	0.30
**Alcohol consumption**			0.25
Absent *n* (%)	8 (100%)	10 (77%)	
Occasional *n* (%)	0 (0%)	1 (8%)	
Regular *n* (%)	0 (0%)	2 (15%)	
Abuse *n* (%)	0 (0%)	0 (0%)	
Mean SBP awake (median [IQR])	131.7 [17.3]	124.8 [18.0]	0.71
Mean SBP sleep (median [IQR])	104.9 [20.1]	123.3 [18.5]	**0.02**
SBP sleep percent change (median [IQR])	14.9 [5.6]	1.7 [7.4]	**< 0.001**
Mean DBP awake (median [IQR])	74.8 [8.7]	70.1 [14.5]	0.24
Mean DBP sleep (median [IQR])	59.4 [5.2]	63.2 [9.4]	0.19
DBP sleep percent change (median [IQR])	22.5 [10.3]	8.4 [8.5]	**0.003**
Aix@75 awake (median [IQR])	25.5 [8.5]	25.0 [10.0]	0.66
Aix@75 sleep (median [IQR])	25.5 [7.5]	36.0 [10.0]	0.13
Aix@75 total (median [IQR])	26.0 [5.3]	31.0 [11.0]	0.26
PWV awake (median [IQR])	10.9 [2.1]	10.9 [0.5]	0.88
PWV sleep (median [IQR])	10.3 [1.7]	10.9 [0.5]	**0.03**
PWV total (median [IQR])	10.6 [1.8]	11 [0.4]	0.36
DTI-ALPS index	1.322 [0.186]	1.146 [0.105]	**0.03**

Significance was tested using the Mann–Whitney *U*-test for continuous variables and Fisher's exact test for categorical variables. Alcohol consumption was coded as follows: absent (no consumption), occasional (not daily intake, < 140 g per week), regular (daily intake, between 140 g and 280 g per week), and abuse (daily intake, more than 280 g per week). BMI, Body mass index; SBP, systolic blood pressure; IQR, interquartile range; MMSE, mini–mental state examination; RBANS, Repeatable Battery for the Assessment of Neuropsychological Status; CDR, Clinical Dementia Rating; AD, Alzheimer's disease; DBP, diastolic blood pressure; Aix@75, augmentation index corrected for a heart rate of 75 bpm; PWV, pulse wave velocity; DTI-ALPS, diffusion tensor imaging along perivascular spaces. Significant differences (*p* < 0.05) are shown in bold.

### DTI-ALPS differences between the SBP dipping and non-dipping groups

**Figure 1:**
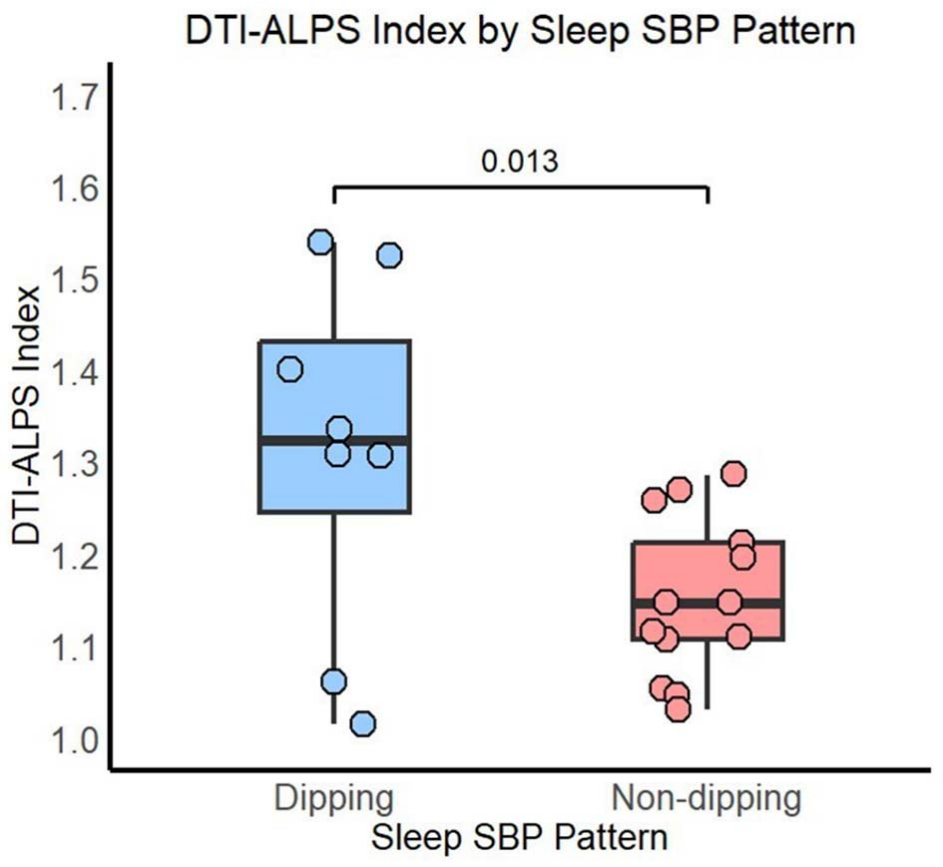
DTI-ALPS index by sleep SBP pattern. *P*-value adjusted for age and the CDR.

Significant differences in DTI-ALPS indices were observed between the dipping and non-dipping groups using the Mann–Whitney *U*-test (*p* = 0.034, ε^2^ = 0.18 CI 95% [0.05, 0.70]]. These differences persisted even after adjusting for age and the CDR in rank-based regression analysis [*p* = 0.013, b = 1.15 CI% [0.33, 1.97]] (see Figure 1). Since the groups differed in years of education, an additional sensitivity analysis including education as a covariate was performed, and the results remained unchanged [*p* = 0.015, b = 1.2 CI% [0.34, 2.1]]. Similarly, as three participants from the non-dipping group reported occasional or regular alcohol consumption, we repeated the analysis after excluding them and still observed significant differences in DTI-ALPS between the SBP dipping (*n* = 8) and non-dipping (*n* = 10) groups [*p* = 0.032, b = 1.1 CI% [0.19, 2.0]].

### Associations between the continuous sleep BP percentage of change and DTI-ALPS

**Figure 2:**
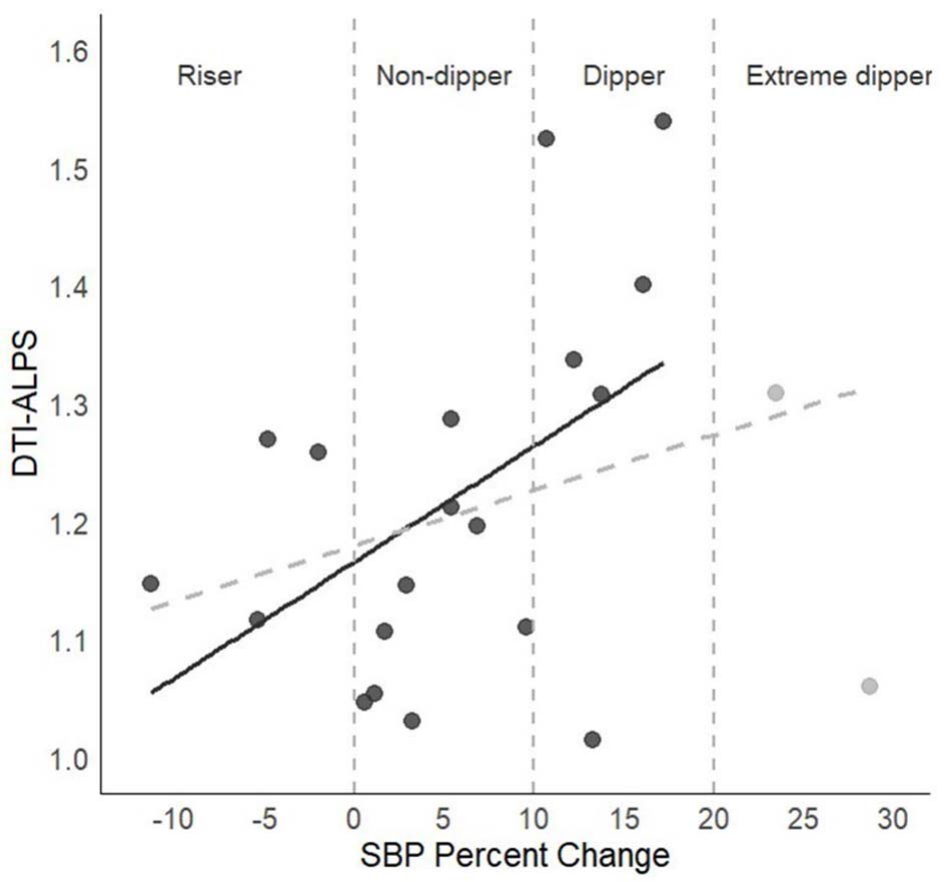
DTI-ALPS index and sleep SBP percentage of change. The dashed gray line represents the association in the full sample, and the black continuous line shows the association after removing the two individuals labeled as extreme dippers.

The correlational analyses in the full sample revealed low positive associations between DTI-ALPS and the percentage of SBP change between awake and sleep, which did not reach statistical significance (*r* = 0.36, *p* = 0.11). After removing extreme dippers (*n* = 19), the associations became moderate and significant (*r* = 0.49, *p* = 0.03; see Figure 2). For DBP, the results were similar (full sample *r* = 0.34, *p* = 0.12; restricted sample *n* = 16, *r* = 0.57, *p* = 0.024).

### Secondary exploratory analyses of normal and abnormal sleep SBP/DBP pattern groups

**Figure 3:**
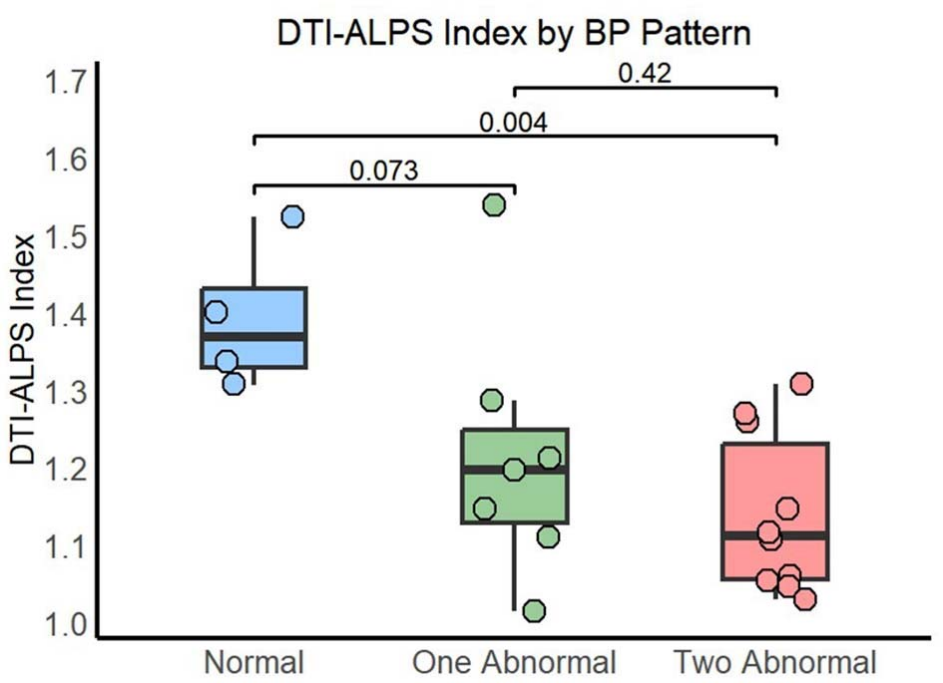
DTI-ALPS index by sleep BP pattern. Normal: dipping pattern in both SBP and DBP; One Abnormal: abnormal pattern in either SBP or DBP; Two Abnormal: abnormal pattern in both SBP and DBP.

A total of four participants (19%) of the 21 recruited displayed normal (dipping) sleep BP patterns in both SBP and DBP. The remaining 17 participants (81%) had abnormal patterns, with the most prevalent group being that with altered patterns in both SBP and DBP (10 participants of 21, 47.6%). The three groups (Normal, One Abnormal, and Two Abnormal) differed in the percentage of participants with positive AD pathophysiological biomarkers (*p* = 0.043) and in the DTI-ALPS index [*p* = 0.024, ε^2^ = 0.31 IC 95% [0.03, 0.67]]. Pairwise comparisons showed that the difference in the percentage of AD-positive biomarkers was driven by the Two Abnormal group compared to the Normal group (*p* = 0.041; One Abnormal vs. Normal *p* = 0.24). Similarly, the DTI-ALPS index was significantly lower in the group with Two Abnormal BP patterns compared to the Normal group [*p* = 0.004, *r*_rb_ = 0.95 IC 95% [0.81, 0.99]] and showed a trend when the One Abnormal group was compared to the Normal one [*p* = 0.073, *r*_rb_ = 0.71 IC 95% [0.15, 0.93]], see Figure 3. The significant difference between the Two Abnormal BP patterns group and the Normal group persisted after adjusting for age [*p* = 0.005, b = 1.50 CI 95% [0.59, 2.40]].

### Associations between stiffness measures, BP groups, and DTI-ALPS

**Figure 4:**
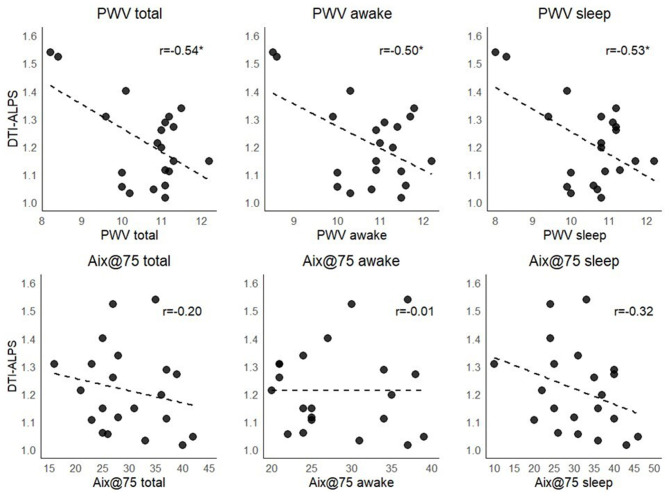
Unadjusted associations between the DTI-ALPS index stiffness measures. PWV, pulse wave velocity; Aix@75, augmentation index corrected for a heart rate of 75 bpm. ^*^p < 0.05.

We observed higher PWV values during sleep in the non-dipping group than in the dipping group [*p* = 0.03, ε^2^ = 0.19 IC 95% [0.05, 0.55]], and this association was moderated when adjusted for age in a rank-based regression analysis [*p* = 0.08, b = 0.52 CI 95% [−0.04, 1.1]]. The correlational analyses between DTI-ALPS and PWV showed significant moderate negative correlations during both awake and sleep periods (around *r* = −0.5, see Figure 4); however, after adjusting for age, these associations disappeared (*p* > 0.4). Regarding Aix@75, no significant associations were observed.

## Discussion

In this study, we investigated the associations between sleep BP patterns and glymphatic function, measured using the DTI-ALPS index, in individuals with cognitive impairment. Consistent with our hypothesis, the findings suggest that deviations from the physiological dipping BP pattern during sleep are associated with reduced glymphatic function, as reflected by lower DTI-ALPS indices.

The main result of this study showed that SBP non-dipping patterns were associated with lower DTI-ALPS indices. This finding persisted even after adjusting for age, education, and clinical stage (CDR). In the secondary exploratory analyses, when any deviation from the physiological dipping pattern was considered (either in SBP or DBP), we also found a higher prevalence of AD pathology, as measured by *in vivo* biomarkers, in the individuals with abnormal patterns. Globally, our findings are in line with the body of literature supporting the relevance of vascular dynamics in glymphatic function. The glymphatic system relies on CSF transport through perivascular spaces, which is driven by arterial pulsatility, respiration, and slow vasomotion (Rasmussen et al., 2022). Altered vascular dynamics, such as reduced arterial compliance and increased peripheral resistance, may contribute to impaired glymphatic clearance, thereby increasing the accumulation of neurotoxic proteins such as Aβ and tau. Evidence from animal models has shown that CSF flow in perivascular spaces is primarily driven by arterial pulsations linked to the cardiac cycle and that under conditions of elevated blood pressure, the efficiency of this arterial-driven CSF flow is reduced (Mestre et al., 2018). Similarly, (Mortensen et al. 2019) demonstrated that in spontaneous hypertensive rats, glymphatic transport is compromised and solute clearance from the brain parenchyma is decreased, with this effect being more pronounced in states of chronic hypertension.

It is well-established that glymphatic transport occurs mainly during sleep (Xie et al., 2013), and it has also been well-documented that BP non-dipping patterns are associated with an increased risk of cognitive decline and dementia (Tan et al., 2021; Gavriilaki et al., 2023). Our hypothesis suggests that the negative outcomes observed in the presence of abnormal sleep BP patterns would act through glymphatic alterations and impaired solute clearance. Fultz et al. showed that during non-rapid eye movement sleep in healthy young humans, large oscillations in CSF flow are tightly coupled with neural and hemodynamic rhythms (Fultz et al., 2019). Specifically, CSF flow oscillations are anti-correlated with hemodynamic oscillations in the cortical gray matter that occur during sleep, with CSF flow increasing as blood volume decreases. Our findings of reduced DTI-ALPS indices in cognitively impaired older individuals with abnormal sleep BP patterns can be interpreted in the light of such observations: the observed altered hemodynamic oscillations at nighttime may, in turn, disrupt CSF flow oscillations, impairing glymphatic function as measured by DTI-ALPS. Therefore, our data support for the first time the concept that cognitive decline associated with the absence of physiological blood pressure drop during sleep may act through glymphatic dysfunction.

The association that we observed between the percentage of BP dip during sleep and DTI-ALPS further supports the concept that a greater nocturnal BP dip is linked to better glymphatic function. However, this association appears to be true only within a certain range of BP change, since the correlations strengthened and became significant (from *r* = 0.3 to *r* = 0.5) after excluding individuals with an extreme-dipping pattern (>20%). Extreme dipping has also been linked to the presence of cognitive impairment (Guo et al., 2010). This evidence, together with our findings, supports the hypothesis that any deviation from the physiological sleep BP dipping range may adversely affect glymphatic function. Moreover, in line with the broader hypothesis suggesting that any alteration in sleep vascular fitness may impair glymphatic function, we observed a negative association between stiffness measures (PWV) and DTI-ALPS. Although the associations disappeared after adjusting for age, our observations align with previous reports linking PWV to an increased risk of dementia (Pase et al., 2016; Heffernan et al., 2024) and greater amyloid deposition (Hughes et al., 2014, 2018). Our results also align with recent evidence showing that PWV is related to enlarged perivascular spaces (ePVS) (Kinjo et al., 2024). ePVS measures have been proposed as another neuroimaging proxy for glymphatic function, since PVS enlargement may occur due to CSF stagnation caused by the disruption of CSF flow.

Although no differences in the frequency of positive AD biomarkers were found when we compared the dippers and non-dippers based on SBP, a higher percentage of individuals with altered AD biomarkers was observed among the participants with abnormal BP patterns in both SBP and DBP, when any deviation from the dipping pattern was considered. This finding aligns with the hypothesis that abnormal BP patterns impair protein clearance, increasing Ab pathology, and mirrors the findings reported by (Tarumi et al. 2015), who observed similar associations using amyloid PET data in MCI patients, focusing on SBP measures. To the best of our knowledge, no other studies have analyzed the relationship between sleep BP patterns and AD pathological markers. Further studies are needed to elucidate the underpinnings of such associations. A study exploring other neuroimaging outcomes demonstrated that increased 24-h AMBP pulse pressure was associated with DTI metrics, such as decreased fractional anisotropy and increased mean diffusivity, suggesting a deterioration in brain neuronal fiber integrity (Tarumi et al., 2019). However, in that study, no association between nocturnal BP dipping and DTI metrics was found. A more recent MRI study performed in a large Korean cohort of nearly 1,400 healthy individuals found that increased systolic or diastolic BP variability during the night, but not mean BP, was associated with reduced temporal gray matter volume and cognitive decline after 4 years. However, the study did not specifically assess BP dipping patterns (Yu et al., 2022).

Night BP pattern alterations may not be solely driven by hypertension or vascular risk factors. The early neurodegeneration of the locus coeruleus (LC), as seen in AD and some alpha-synucleinopathies, may also contribute to abnormal sleep BP patterns. The LC, in addition to its known regulation of the sleep-wake cycle, also innervates brain precapillary and capillary vessels, regulates neurovascular coupling, and controls astrocytes, endothelial cells, and pericytes. These components together constitute the neurovascular unit, which is central to the functioning of the glymphatic system (Giorgi et al., 2020). Indeed, the magnitude of the circadian pattern of BP, but not overall BP levels, has been shown to decrease after LC lesions in rats (Mitsubayashi et al., 1995; Mitsubayashi, 2000). The fact that in AD, the LC starts accumulating p-Tau decades before the onset of cognitive impairment (Braak et al., 2011), and that in Parkinson's disease, LC disintegration is related to dysautonomia (Madelung et al., 2022), which is associated with faster disease progression (De Pablo-Fernandez et al., 2017), suggests a relevant role of the link between the LC and circadian BP in the etiology and progression of neurodegenerative diseases. In a previous study, we presented a model linking glymphatic dysfunction to the progression of alpha-synucleinopathies, emphasizing the role that vascular regulation disturbances may play in the heterogeneous evolution of such diseases (Buongiorno et al., 2023). The current findings further support this model by suggesting that abnormal sleep BP patterns significantly reduce glymphatic efficiency, as measured by DTI-ALPS. Our model proposed that glymphatic dysfunction, synergistically enhanced by sleep disturbances and vascular dysregulation, fosters a vicious cycle of protein accumulation and neurodegeneration. The observed association between abnormal BP patterns and reduced DTI-ALPS indices in the present study strengthens the argument that vascular dynamics, regulated by the LC, are crucial for maintaining glymphatic efficiency. Importantly, this proposal expands the etiology of BP abnormalities beyond purely vascular risk factors (i.e., hypertension), suggesting that very early LC degeneration may serve as a hypothetical starting point for protein accumulation by impairing circadian BP regulation.

Our findings may have therapeutic implications and encourage further research into sleep-time BP control as a potential enhancer of glymphatic clearance. If abnormal nocturnal BP profiles, specifically a non-dipping pattern, are linked to poorer glymphatic function as measured by DTI-ALPS and, by extension, to reduced protein clearance, then restoring a normal dip may help prevent or slow neurodegenerative disease progression. Systematic reviews indicate that taking antihypertensives at bedtime reduces the proportion of patients who remain non-dippers (Lee et al., 2024). Similarly, a recent randomized trial showed that moving a once-daily olmesartan/amlodipine dose to bedtime reduced nighttime systolic BP by 3 mm Hg, raised nocturnal SBP control to 79%, and reduced the non-dipping rate from 53.3% to 36.9% over 12 weeks, while morning dosing produced no comparable change (Ye et al., 2025). Prospective studies are needed to test whether such chronotherapy improves glymphatic proxies, lowers AD risk, and impacts clinical progression.

The present study has some limitations. The primary ones include the relatively small number of participants recruited, the cross-sectional nature of the study, and the lack of a control group without cognitive impairment. However, the participants were well-characterized, with available AD biomarker status. Regarding neuroimaging methods, we acknowledge the limitations of the DTI-ALPS index as a measure of glymphatic function (Ringstad, 2024). However, it has been widely used as a feasible glymphatic proxy, and it correlates with intrathecal administration of gadolinium (*r* = 0.84), which is considered the gold standard method for assessing glymphatic function (Zhang et al., 2021). Another limitation is that the sample was heterogeneous in terms of vascular risk factors and the presence of hypertension. In addition to using the standard methods to define dipping and non-dipping patterns based on the commonly used SBP percentage of dip (< 10%), we also categorized the participants into Normal, One Abnormal, and Two Abnormal BP groups by combining SBP and DBP patterns. Although this approach is non-standard and we acknowledge that SBP and DBP are highly correlated, we performed secondary analyses using these groups to account for any deviations from the normal sleep vascular BP pattern, given the exploratory nature of the study. Finally, we acknowledge that the lack of adjustment for multiple comparisons in the secondary exploratory outcomes may have inflated the probability of Type I errors. Therefore, further studies with larger sample sizes and adequate statistical power are needed to confirm the observed associations, which should be considered preliminary. On the other hand, this study has several strengths. It is the first to address the association between sleep BP patterns and MRI glymphatic proxies, and it was conceptualized within a broader theory of the interplay between the glymphatic system and its vascular drivers. Although some associations were observed, further longitudinal studies with larger samples are needed to confirm these findings.

In conclusion, our findings suggest that deviations from the physiological dipping sleep BP pattern in cognitively impaired individuals are associated with lower DTI-ALPS measures, a proxy of glymphatic dysfunction. Abnormal BP patterns are also related to the presence of AD pathological biomarkers. These results imply that nighttime BP alterations may impact the functioning of the glymphatic system, possibly contributing to pathological processes in AD. Although the generalizability of our results is limited due to the small sample size and the cross-sectional nature of the study, and replication in larger samples is needed, these findings open new avenues for research into therapeutic strategies targeting vascular health—such as antihypertensive treatments promoting physiological BP dipping during sleep—which could have significant implications for slowing or preventing the progression of neurodegenerative diseases.

## Data Availability

The raw data supporting the conclusions of this article will be made available by the authors, without undue reservation.
